# Supplementary material for: Comparative Effects of Particle Sizes of Cobalt Nanoparticles to Nine Biological Activities
Source: Int J Mol Sci. 2020 Sep 15;21(18):6767. doi: 10.3390/ijms21186767 (PMC7555351; doi:10.3390/ijms21186767)
Supplement: Supplementary file 1 [file ijms-21-06767-s001.pdf]

Supplementary Materials

Table 1. Comparisons of the toxicity values of the different sizes of Co-NPs: (A) toxicity on the revertant mutation, (B) average toxicity for two endpoints of algal growth.

(A)			(B)		
Experimental conditions	Toxicity (%)		Experimental conditions	Average toxicity (%)	
	100 mg/L	500 mg/L		1000 mg/L	2000mg/L
type A	35 ± 12.7	25 ± 1.9	type A	85 ± 8.5	89 ± 3.7
type B	−20 ± 4.1	−29 ± 7.1	type B	40 ± 9.7	77 ± 15.3

Table 2. Summary of two doses tested for each bioassay.

#	**Experiment**	**Co-NP (mg/L)**	#	**Experiment**	**Co-NP (mg/L)**
1	Bioluminescence assay	100, 200	4	Gene mutation assay	100, 500
2	Bioreporter strain activity assay	5, 10	5	Algal growth assay	1000, 2000
3	Activity and biosynthesis of β-galactosidase	200, 600	6	Seed germination, root, and shoot growth assay	1000, 2000
